# Thermodynamic interference with bile acid demicelleization reduces systemic entry and injury during cholestasis

**DOI:** 10.1038/s41598-020-65451-w

**Published:** 2020-05-21

**Authors:** Cristiane de Oliveira, Biswajit Khatua, Bara El-Kurdi, Krutika Patel, Vivek Mishra, Sarah Navina, Bradley J. Grim, Srishti Gupta, Marek Belohlavek, Brian Cherry, Jeffery Yarger, Matthew D. Green, Vijay P. Singh

**Affiliations:** 1 Department of Medicine, Mayo Clinic, Scottsdale, AZ USA; 2 Department of Medicine, University of Pittsburgh, Pittsburgh, PA USA; 3 Department of Pathology, University of Pittsburgh, Pittsburgh, PA USA; 4 Department of Chemical Engineering, School for Engineering of Matter, Transport and Energy, Arizona State University, Tempe, AZ USA; 5 Department of Cardiovascular Medicine, Mayo Clinic Arizona, Scottsdale, USA; 6 School of Molecular Sciences, Arizona State University, Tempe, AZ USA; 7 Department of Biochemistry and Molecular Biology, Mayo Clinic Arizona, Scottsdale, USA

**Keywords:** Biophysical chemistry, Blood flow

## Abstract

Bile acids (BA), with their large hydrophobic steroid nucleus and polar groups are amphipathic molecules. In bile, these exist as micelles above their critical micellar concentration (CMC). In blood at low concentrations, these exist as monomers, initiating cellular signals. This micellar to monomer transition may involve complex thermodynamic interactions between bile salts alone or with phospholipids, i.e. mixed micelles and the aqueous environment. We therefore went on to test if therapeutically relevant changes in temperature could influence micellar behavior of bile salts, and in turn whether this affected the biological responses in cells, and *in vivo*. Sodium taurocholate (STC) belongs to a major class of bile salts. STC has a CMC in the 5–8 mM range and its infusion into the pancreatic duct is commonly used to study pancreatitis. We thus studied micellar breakdown of STC using isothermal titration calorimetry (ITC), dynamic light scattering and cryogenic transmission electron microscopy. Under conditions relevant to the *in vivo* environment (pH 7.4, Na 0.15 M), ITC showed STC to have a U shaped reduction in micellar breakdown between 37 °C and 15 °C with a nadir at 25 °C approaching ≈90% inhibition. This temperature dependence paralleled pancreatic acinar injury induced by monomeric STC. Mixed micelles of STC and 1-palmitoyl, 2-oleyl phosphatidylcholine, a phospholipid present in high proportions in bile, behaved similarly, with ≈75% reduction in micellar breakdown at 25 °C compared to 37 °C. *In vivo* pancreatic cooling to 25 °C reduced the increase in circulating BAs after infusion of 120 mM (5%) STC into the pancreatic duct, and duct ligation. Lower BA levels were associated with improved cardiac function, reduced myocardial damage, shock, lung injury and improved survival independent of pancreatic injury. Thus micellar breakdown of bile salts is essential for their entry into the systemic circulation, and thermodynamic interference with this may reduce their systemic entry and consequent injury during cholestasis, such as from biliary pancreatitis.

## Introduction

Severe cholestasis from biliary obstruction can be associated with an inexplicable and rapid transition to shock, organ failure and early mortality during acute stress^1–7^. While this has been known for several decades^8^, and is debated (e.g. whether or when to drain bile before surgery, chemotherapy or during biliary pancreatitis), the reasoning underlying this is so-far unclear. Critically ill patients with elevated bile acids have higher mortality^9^, and cholestasis from a sterile diseases such as a malignancy can result in mortality^1^, organ failure^2,3^ and increase post- operative mortality^10^. Such observations may have shaped medical and surgical practice^2,4,11^, such as when and whether to operate, or give chemotherapy. However the mechanisms underlying these can be clarified further.

Bile salts may play a mechanistic role in the above phenomena since these are the most prevalent class of molecules in bile (50–200 mM)^12^, and bile acids are present in human pancreatic necrosis collections^13^. Bile also contains the phospholipid phophatidylcholine with unsaturated or mixed long chain fatty acids at the sn-1, sn-2 position^14,15^. The bile salt: phospholipid ratios in bile range from 3:1^16,17^ to 15:1^18,19^. Interestingly, while the common channel hypothesis has been questioned^20^, low pressure STC infusion into the pancreatic duct is commonly used to model severe pancreatitis^21,22^, with numerous publications using it (https://pubmed.ncbi.nlm.nih.gov/?term=sodium+taurocholate+AND+pancreatitis+AND+duct.) over the last 40 years since its inception. Moreover recent studies have shown that bile salts are present in human pancreatic necrosis collections^13^. We therefore used a combination of STC infusion under low pressure, followed by ligation of the biliopancreatic duct, as shown previously^23^, to allow reflux of bile and the phospholipids it contains at native pressures in the pancreatico-biliary duct system. Recent studies show that local pancreatic cooling via a gastric balloon can reduce the severity of lipotoxic and caerulein pancreatitis^24^. In this study we tested the efficacy of this in affecting local and systemic injury in the model of biliary pancreatitis described above.

Bile salts have a large steroid nucleus forming a rigid hydrophobic discoid structure on which 1 to 3 hydroxyl groups face the concave aspect^25^. The hydrophobic area contributes to the back to back^25^ self-association of BAs^26^, shielding these from water^27^ and forming dimers of primary micelles, while the intermolecular hydrogen bonding of the hydroxyl groups results in secondary micelles^28^. Di- and trihydroxy BAs comprise >98%^29,30^ of bile acids, and CMC of BAs increases with the number of hydroxyl groups, being <1 mM for monohydroxy BAs^31^ and >5 mM for the tri-hydroxy ones^28^. This is relevant to the way in which BAs may enter the circulation or signal, which is possible in 2 ways. (1) via receptors (e.g. FXR or gpBAR1^32^) which are activated in the micromolar range, below the CMCs and therefore likely by monomeric BAs, and 2) via membrane damage^33^ occurring above their critical micellar concentrations such as during hemolysis^33^, a bile leak, or reflux of bile into the pancreas^21,22^. Since BA levels in circulation are in the sub-micellar range^6,7^, we hypothesized that breakdown of bile salt or mixed micelles following STC pancreatitis would be essential for their entry into the circulation and mediating systemic effects. Therefore, we proposed to study whether interference with the micellar breakdown may be beneficial in STC induced pancreatitis followed by duct ligation.

Previous studies have used isothermal titration calorimetry (ITC) to study bile salt micellar breakdown^34–36^. ITC allows us to directly study demicellization enthalpy (ΔH) as a function of temperature. This is especially important since CMC itself may change with temperature^29,37^, and our intent was to impede micellar breakdown by varying temperature. We thus chose ITC to study the thermodynamics of BA micelle breakdown. The conditions used were relevant to those of the infused STC and circulating bile acids (pH 7.4 and 0.15 mM Na), which helped identify the temperatures that interfere with micellar breakdown and could potentially be applied as local hypothermia during these diseases. Acute pancreatitis has the most rapid onset and progression among the various diseases. Thus as mentioned above, we chose to test the effect of cooling in a severe pancreatitis model, by infusing STC into the pancreatic duct^21,22^ followed by ligating the biliopancreatic duct^23^ to allow passive reflux of bile and the phospholipids it contains into the pancreatic duct. We then studied whether achieving pancreatic temperatures that interfere with breakdown of STC micelles, and mixed STC+ phospholipid micelles can change the disease course. This study describes the therapeutic relevance of preventing bile salt demicellization on disease severity.

## Results

### Thermodynamic interference impairs breakdown of simple bile salt micelles

**Figure 1:**
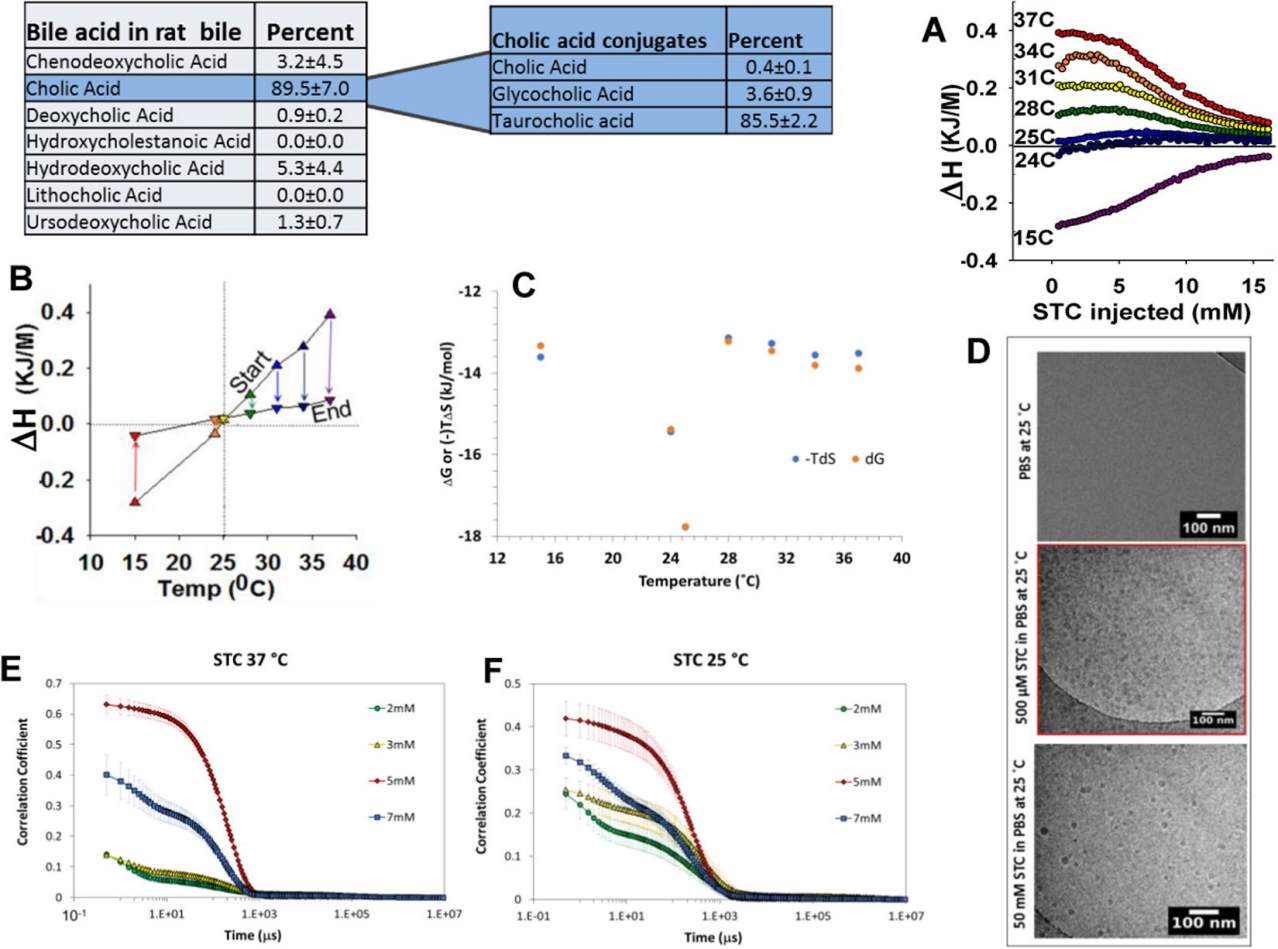
Transition of endothermic micellar breakdown of a high prevalence BA to exothermic with cooling, and cessation of micellar breakdown of STC at 25 °C. Table showing the concentrations of bile acids (left) and Cholic acid conjugates (Right, dark blue background) measured by LC MS/MS in the bile of rats **(A)** Enthalpograms representative of micellar breakdown of STC at the indicated temperatures. Note the progressive reduction in magnitude of endothermic reaction enthalpy (ΔH) to exothermic at 15 C. This is shown as an average of 3 experiments. (**B)** Change in enthalpy (ΔH) from the initiation of micellar breakdown (start, upward triangles) till equilibrium was reached (end, downward triangles). Note that the two line intersect at 25 C (yellow star) and that the equilibrium becomes progressively exothermic, supporting the contribution of hydroxyl groups in stabilizing micellar structure. **(C)** The Gibbs free energy and entropy of the titration curves at various titration temperatures. The minimum at 25 °C indicates favorable conditions for STC micelles, which correspond to a near athermal titration and a large reduction in CMC. (**D)** Cryo-TEM images showing PBS and solutions of STC at 50 mM and 500 µM in PBS analyzed at 25 °C. Note the presence of micelles at both STC solutions. Autocorrelation functions collected from STC at 7 mM (blue), 5 mM (red) 3 mM (yellow), and 2 mM (green) in PBS at 37 °C (**E**) and 25 °C (**F**).

Our studies started with the sodium salt of the trihydroxy BA, cholic acid, i.e. sodium taurocholate (STC), since cholic acid is the predominant bile acid in rodents [85 ± 0.5% of bile acids in rats (Fig. 1 table)] and humans^30,38,39^, in both of whom it is mainly conjugated to taurine (Supplementary Fig. 1). Moreover STC is commonly used to induce biliary pancreatitis^21^. The initial studies were done using simple STC micelles, to retain relevance to the commonly used model of pancreatitis induced by STC infusion into the pancreatic duct^21,22^.

**Figure 2:**
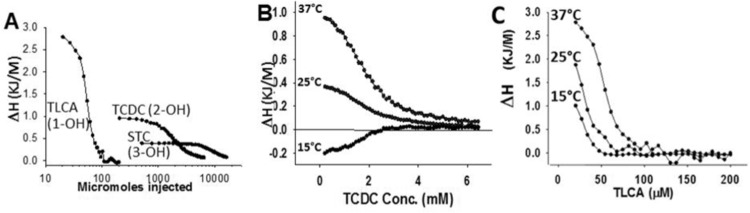
Influence of polar groups and temperature on micellar breakdown: (**A**) Enthalpograms of micellar breakdown at 37 C obtained with injection of the sodium salts of the monohydroxy (1-OH) bile acid taurolithocholic acid (TLCA), the di-dyroxy (2-OH) bile acid taurodeoxy cholic acid (TCDC) and the tri-hydroxy (3-OH) bile acid sodium taurocholate (STC) into PBS (pH, 7.4, containing 150 mM Na). Note the reduction in reaction enthalpy with the increase in hydroxyl groups. Representative enthalpograms showing the change in enthalpy of TCDC (**B**) and TLCA (**C**) with dilution from stock solutions of 20 mM and 1 mM respectively made in PBS. Note lowering of CMC in TLCA along with the more endothermic enthalpy which is less responsive to cooling, implying that the hydroxyl groups contribute to an increasingly exothermic micellar breakdown with cooling.

During ITC, When STC was injected from a 50 mM stock (in PBS, pH 7.4 at 37 °C, which is 6–10x CMC) into PBS at concentrations below CMC, there was a dose dependent heat uptake with STC dilution, until concentrations near CMC (≅5 mM) were reached (Supplementary Fig. 2A). After this, the heat rate decreased with consecutive injections implying a progressive reduction in micellar breakdown. This phenomenon–an increase in heat uptake–was not seen when the STC stock was below the CMC (i.e. 3.4 mM, at 37 °C); the heat rate being the same as for an injection of PBS into PBS (Supplementary Fig. 2A top 2 tracings). Before reaching the CMC, the heat recorded had a linear relation to the concentration of STC injected (Supplementary Fig. 2B, table). We then measured the effect of cooling on micellar breakdown at temperatures relevant to the *in vivo* environment. Cooling from 37 to 25 °C, dose dependently reduced the magnitude of reaction enthalpy associated with micellar breakdown (Fig. 2A). The corrected heat rate reduced from 101.5 μJ/s at 37 °C (254 μmol/injection from a 50 mM stock) to 12.3 μJ/s at 25 °C (Supplementary Fig. 2C). This became exothermic at 15 °C (−79 μJ/s). The heat rate of injecting 50μM from a 3.4 mM stock of STC at 25 °C remained the same as at 37°C (Supplementary Fig. 2D). Since the volume, concentrations, and pressure at all temperatures were the same, the entropy of this system could only be affected by changes in random motion due to cooling and altered interactions with the solvent. Interestingly, at concentrations above 15 mM (2–3x of CMC), the heat of equilibrium transitioned from endothermic at 37 °C to exothermic at 15 °C (Fig. 1B, Supplementary Fig. 3A,B), with no detectable difference in the enthalpy from start to finish in the titration at 25 °C. The derivative of the enthalpy with respect to concentration shows the location of the inflection point for the enthalpy titration curves (Fig. 1A), which reveals the CMC at that temperature. At temperatures from 28–37 °C as well as 15 °C the CMC remains relatively stable around ~8 mM, However, around 25 °C the CMC reduced dramatically, approaching 0 mM (Supplementary Fig. 3C). These values for CMC were used to calculate the ΔG according to the mass action model, which subsequently allowed for the calculation of TΔS (Fig. 1C, methods described in the Supplementary Information). Collectively, these data indicate a thermal titration behavior around 25 °C, which yields a free energy minimum and makes micellar breakdown at 25 °C highly unfavorable. However, micellar breakdown is possible at temperatures above and below this narrow window.

This data was further tested by performing dynamic light scattering (DLS), and cryogenic transmission electron microscopy (cryo-TEM) experiments. DLS was performed at 37 °C and 25 °C in PBS with STC concentrations of 2–7 mM. The autocorrelation functions (Fig. 1E,F) are shown rather than size distribution plots due to the polydisperse nature of the STC aggregates. Several interesting features in the autocorrelation plots were observed. First, increasing concentration from 5 mM (the reported CMC for STC) to 7 mM at both temperatures caused a decrease in the correlation coefficient intensity and the appearance of a shoulder at short decay times. We attribute this to multiple particle scattering at these higher concentrations. Next, decreasing concentration from 5 mM to 3 mM and 2 mM had a markedly different impact on the auto correlation function at 37 °C versus 25 °C. At 37 °C, decreasing the concentration below the CMC caused a dramatic drop in the correlation coefficient intensity, indicative of a precipitous drop in the ratio of micelles to monomers. However, at 25 °C, decreasing the concentration caused what appears to be a more linear decrease in the correlation coefficient, which signified a linear decrease in micelle concentration corresponding to the decrease in solution concentration. In other words, we propose that the ratio of micelles to monomer did not change at 25 °C (i.e., simply the concentration as a whole decreased). Thus, the DLS data corroborate the findings from ITC in that micellar assemblies remain stable at 25 °C.

Finally, cryo-TEM was performed to visualize the formation of micelles in the STC solutions. Solutions of STC at 50 mM and 500 µM in PBS were analyzed at 25 °C (i.e., solutions were incubated at 25 °C prior to sample preparation, for additional details see the methods section). The micrographs clearly indicate the presence of micelles in both solutions, roughly 15–20 nm in diameter. Again, the shift in CMC and stabilization of micelles below 5 mM at 25 °C was corroborated using cryo-TEM. Thus we noted micellar breakdown of STC to be impeded at 25°C using 3 complementary methods.

Based on previous studies reporting the large hydrophobic steroid nucleus’ role in micellar aggregative behavior, and hydroxyl groups in hydrogen bonding^25–28^, we went on to understand the interplay of hydrophobic effect versus hydroxyl groups in explaining our findings. For this we used the sodium salt of a mono-hydroxy bile acid taurolithocholic acid (TLCA), the sodium salt of a di-hydroxy taurochenodeoxycholic acid (TCDC), and the tri-hydroxy STC. TLCA provides an important tool to study this interplay, even though it forms <1% of the bile acid pool in rodents and humans (Fig. 1 table, Supplementary Fig. 1) and thus would not be relevant to diseases of interest to us.

While micellar breakdown was endothermic for all bile acids at 37 °C (Fig. 2A), the highest enthalpy change was noted for TLCA and the lowest for STC. As the magnitude of enthalpy change decreased with increasing the number of hydroxyl groups, the concentration at which micellar formation occurs increased from <100 μM for TLCA, to ~2 mM for TCDC, to >5 mM for STC. These findings are consistent with previous reports^26,31,37^, that suggest hydrogen bonding to inhibit micellar breakdown. We additionally noted a 60% reduction in reaction enthalpy from 37 °C to 25 °C for TCDC (Fig. 2B), and only a 30% reduction for the TLCA (Fig. 2C) vs. the 88% noted for STC (Fig. 1A,B). Based on the above we can conclude that cooling bile acids in a micellar form allows the hydroxyl groups to stabilize micelles even when they are diluted, which prevents micellar breakdown. However, conversely more hydroxyl groups (e.g. STC) increase hydrogen bonding, which becomes stronger upon further cooling to lower temperatures (e.g. 15 °C) thus driving an exothermic breakdown of micelles. At 25 °C (Supplementary Fig. 2D) and 15 °C however, the intermolecular interactions are vastly stronger than the acid-solvent interactions, preserving the micellar structure. These findings which dictated the temperature range for cell and *in-vivo* studies suggest the temperature dependent balance between two strong intermolecular BA-BA (i.e. hydrophobic and hydrogen bonding), and weaker BA-solvent interactions modulates the stability of micellar BA aggregates.

### Temperatures preventing micellar breakdown reduce bile salt toxicity

**Figure 3:**
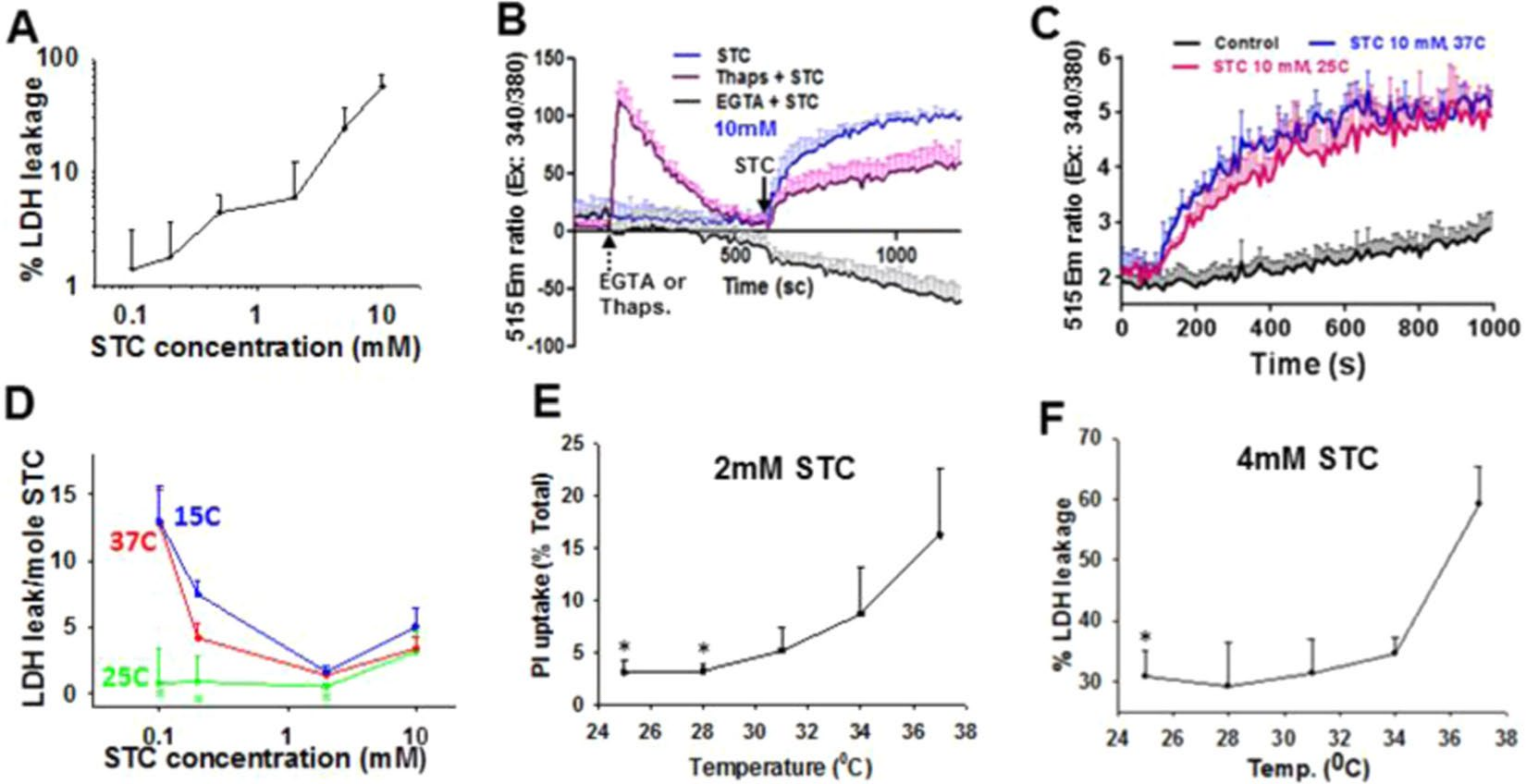
Effect of temperatures altering micellar behavior on cell injury. (**A**) Relationship of STC concentration to the magnitude of cell injury as measured by LDH leakage. (**B,C)** Cytosolic calcium increase induced by supra-micellar (10 mM) STC as measured using Fura-2 AM. (**B)** shows the effect of extracellular calcium chelation with 5 mM EGTA (black line) or depletion of intracellular calcium stores using thapsigargin (Thaps.; 100 μM) on the cytosolic calcium increase induced by 10 mM STC. Note that the increase is completely prevented by EGTA but not thapsigargin- supporting that STCs effect at 10 mM is via direct membrane damage. (**C)** shows that cooling to 25C does not affect the increase in cytosolic calcium induced by 10 mM STC. (**D)** Dose response curves showing efficacy of STC to induce cell injury at different temperatures (Red; 37 °C, Green 25 °C, blue 15 °C). Note that the injury per mole is higher at sub-micellar concentrations when STC is monomeric, except at 25 °C when we note micellar breakdown to cease. (**E,F)** Effect of graded hypothermia on propidium iodide (PI) uptake at 2 mM (**E**) and LDH leakage at 4 mM (**F**). Note that the protective effect of cooling diminishes with increasing concentrations which supports micellar damage from the increasing concentrations of micelles occurs at 25 °C. *Indicated a p < 0.05 vs. 37C by one way ANOVA.

We went on to test the physiological relevance of our hypothesis in cells. For this we chose primary pancreatic acinar cells that have been shown to respond to sub-micellar concentrations of bile salts via the bile acid receptor GpBAR1^40^. Such reductionist models of exposing acini to bile salts^40–42^ are commonly used to study the pathophysiology of pancreatitis. At 37°C, STC caused cell injury, seen as LDH leakage with increasing concentrations (Fig. 3A). Effects mediated by supra-micellar concentrations (10 mM) were due to membrane damage. This was evidenced by the 10 mM STC (i.e. above CMC) causing cytosolic calcium increase from an extracellular pool, since this was inhibited by the extracellular calcium chelator EGTA (Ethylene glycol-bis(β-aminoethyl ether)-N,N,N′,N′-tetraacetic acid; Fig. 3B), but not thapsigargin (Thaps.) which depletes intracellular calcium pools. This detergent mediated signaling was unaffected by cooling to 25 °C (Fig. 2C). While the injury mediated by monomeric STC (e.g. <2 mM) was smaller in magnitude than STC above CMC at 37 °C (Fig. 3A), it was greater than that induced by STC at concentrations above the CMC on a per mole basis (Fig. 3D, red line). Cooling to 25°C (green line, Fig. 3D) reduced the injury induced by submicellar STC (Fig. 3D–F), while being ineffective above the CMC (Fig. 3C,D). This provides physiologic relevance to the reduction in micellar breakdown of STC we noted at 25°C (Fig. 1) and is supported by a loss of protection by further cooling to 15°C (Fig. 3D, blue line), when exothermic micellar breakdown occurs (Fig. 1, Supplementary Fig. 2C). The statistically significant, but reduced protective effect of cooling that we note with increasing STC concentrations (e.g. 4 mM vs. 2 mM, Fig. 3E,F), suggests that while the monomeric form may be reduced at 25 °C, the resulting increase in micellar from may cause direct membrane damage.

### Mixed STC+ phosphatidylcholine micelles display thermodynamic behavior and acinar cell injury responses similar to simple STC micelles

We next studied the thermodynamic behavior of mixed STC- phosphatidylcholine micelles that would be relevant to human bile^18,19^, and also the commonly used STC model of pancreatitis^21,22^ followed by biliopancreatic duct ligation^23^. For this we used the phospholipid 1-palmitoyl, 2-oleyl phosphatidylcholine (POPC) which is strongly represented in human bile^15,43^. Mixed micelles of STC and POPC were used at a 9:1 ratio based on the biliary concentrations noted previously^18,19^.

**Figure 4:**
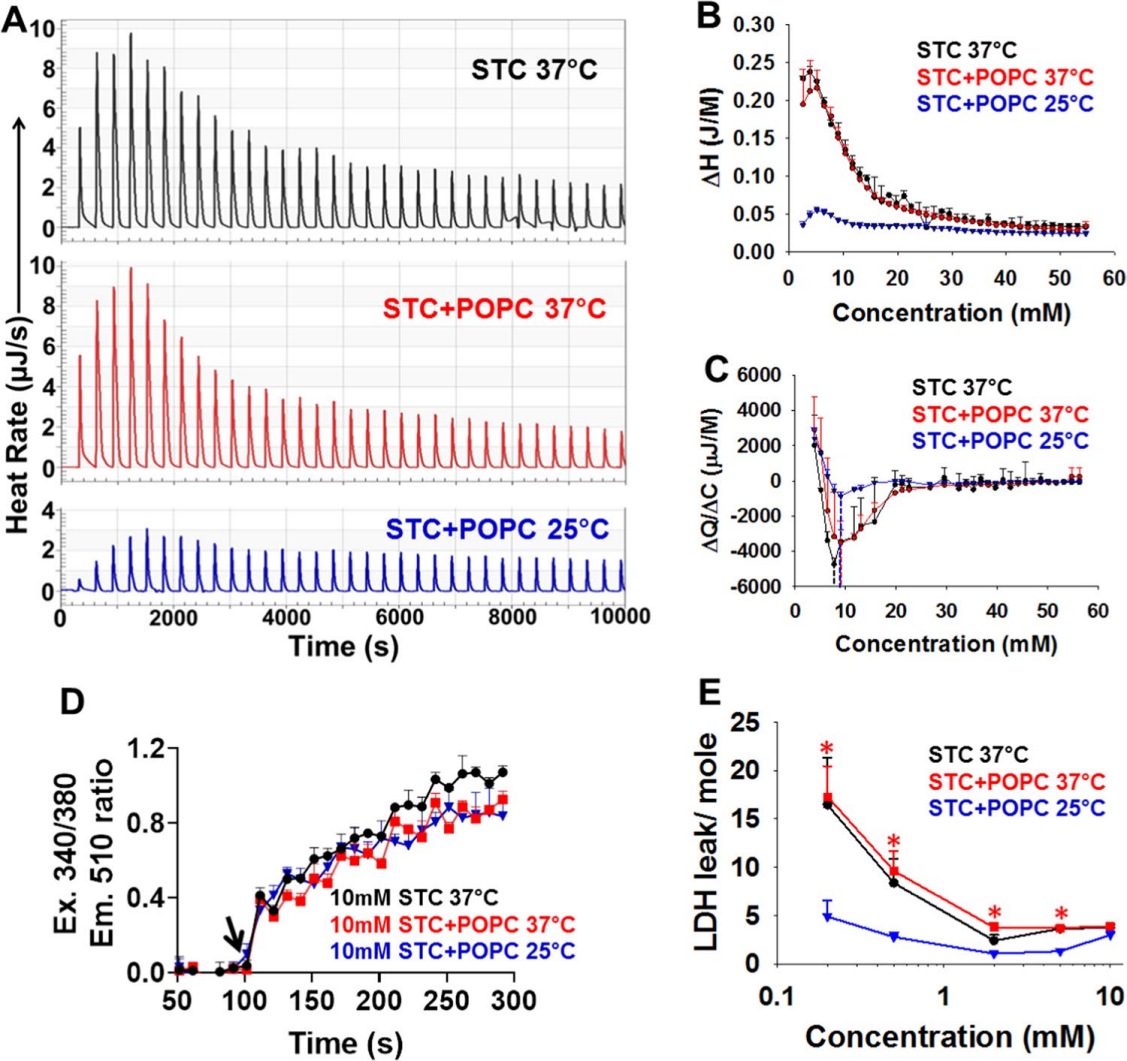
Effect of altering temperatures on micellar behavior and cell injury induced by mixed micelles (9:1, STC:POPC, 1-palmitoyl, 2-oleyl phosphatidylcholine) in comparison to STC alone. (**A**) Thermograms representative of micellar breakdown at the indicated temperatures. (**B)** Enthalpograms representative of micellar breakdown of at the indicated temperatures. Data is from 3 experiments per condition **(C)** Critical micellar concentrations (dashed lines) measured as change in raw heat/ change in concentration (ΔQ/ΔC) at the indicated temperatures. Data is from n=3 experiments per condition (**D)** Cytosolic calcium increase induced by supra-micellar (10 mM) STC or STC + POPC at the indicated temperatures as measured using Fura-2 AM. Data is from 3 experiments per condition (**E**) Dose response curves comparing STC to STC + POPC in inducing cell injury at the indicated temperatures. Note that the injury per mole is higher at sub-micellar concentrations at 37 °C when the agents are monomeric, similar to what we note in Fig. 3D. *Indicated a p < 0.05 vs. 37C by one way ANOVA. Data is from 8 experiments per condition.

The thermodynamic behavior of STC+ POPC was very similar to STC (Fig. 1), with ≈75% reduction in micellar breakdown at 25 °C vs. 37°C (Fig. 4A,B). There was a small and insignificant shift in CMC from 7.4 mM for simple STC to 8.9 mM for STC+ POPC (dashed lines Fig. 4C). Similarly the increase in Cai induced by supramicellar (10 mM) STC+ POPC was similar to that of STC alone (Fig. 4D) and unaffected by cooling to 25 °C. Consistent with the reduction in micellar breakdown at 25 °C mentioned above, There was a reduction in LDH leakage/mole of STC+ POPC micelles. Both the magnitude of injury/mole at 37 °C, and the extent of protection at 25°C reduced with increasing STC+ POPC concentrations (Fig. 4E), as was noted for STC alone (Fig. 3D). Therefore the thermodynamic behavior and acinar cell injury induced by mixed bile salt+phospholipid micelles representative of human bile (and also the STC model of pancreatitis^21,22^ followed by biliopancreatic duct ligation^23^), remained similar to simple STC micelles. Please note saturated phospholipids are a negligible part of gallbladder bile^14,15^. Therefore the previous studies by Moachetta etal showing dipalmitoyl phophatidyl choline reduces bile salt injury^44^ are not relevant to biliary pancreatitis. Interestingly, similar to our data, they showed egg yolk phosphatidyl choline, which contains a large proportion of POPC, had no protective effect from taurocholate induced injury at a 1:3 ratio (Fig. 4 of cited manuscript). Noting the protective effect of preventing micellar breakdown on cell injury, we went on to study its therapeutic relevance *in vivo* by achieving these pancreatic temperatures via a temperature controlled balloon in the stomach as described recently^24^.

### Thermodynamic interference *in vivo* prevents systemic entry and toxicity of bile acids

**Figure 5:**
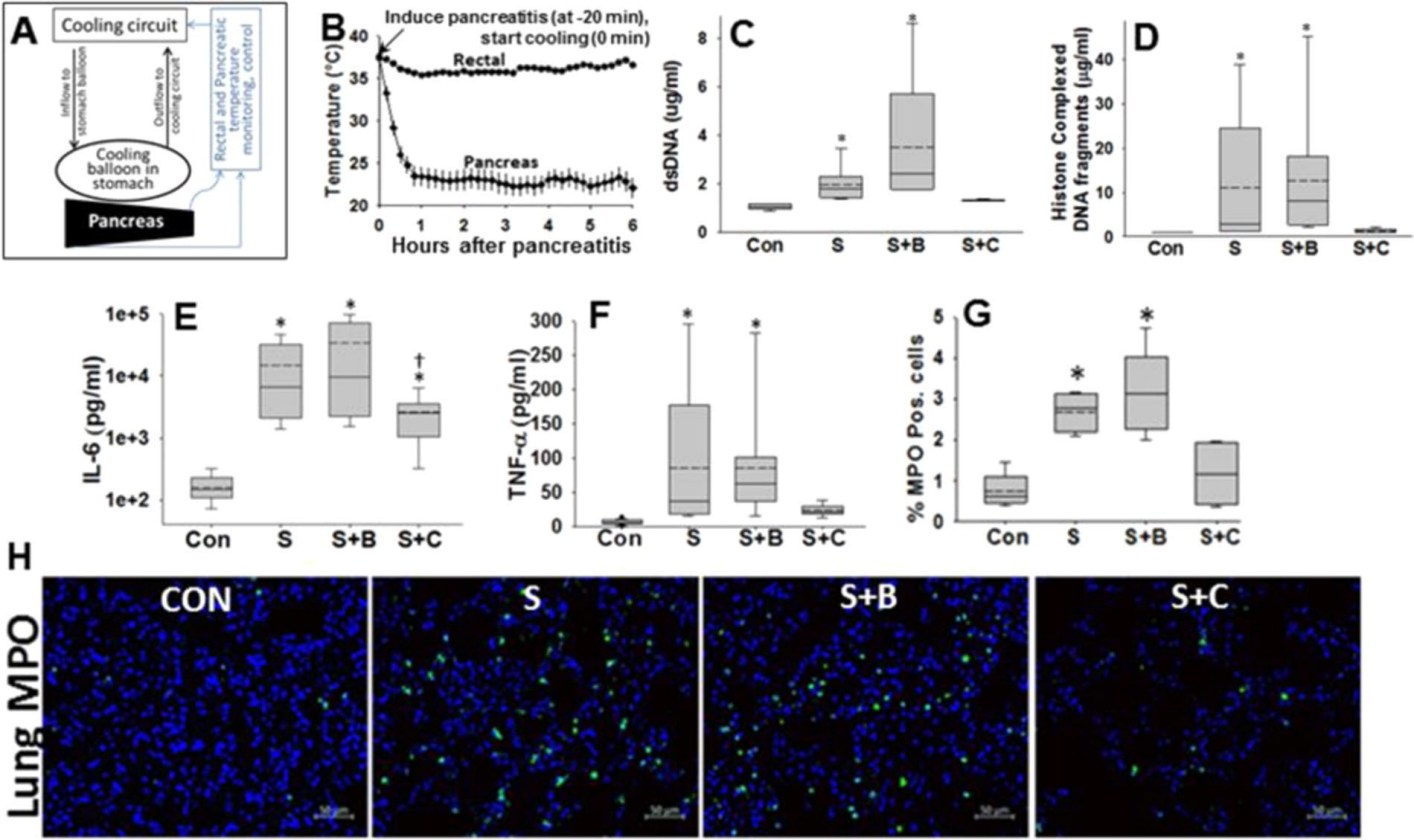
Setup, time line and efficacy of local pancreatic cooling to impair micellar breakdown in reducing systemic injury: (**A**) Schematic showing set up of pancreatic cooling of via perfusion of gastric perfusion. The details are described in methods. (**B)** Timeline and temperature recordings from thermocouple probes in the rectum and pancreas (averaged for the head and tail probes). Box plots showing plasma levels of dsDNA (**C**), histone complexed DNA fragments (**D**), Serum IL-6 (**E**), TNF-α (**F**), and percentage of myeloperoxidase (MPO) positive cells in the lungs (**G**) in controls (Con), STC infused groups without balloon (S), with just balloon and no cooling (S + B) and Balloon with cooling (S + C). *Indicate a significant difference from controls on one-way ANOVA. (**H)** shows representative lung images from each group of MPO positive cells (green) and DAPI positive nuclei.

**Figure 6:**
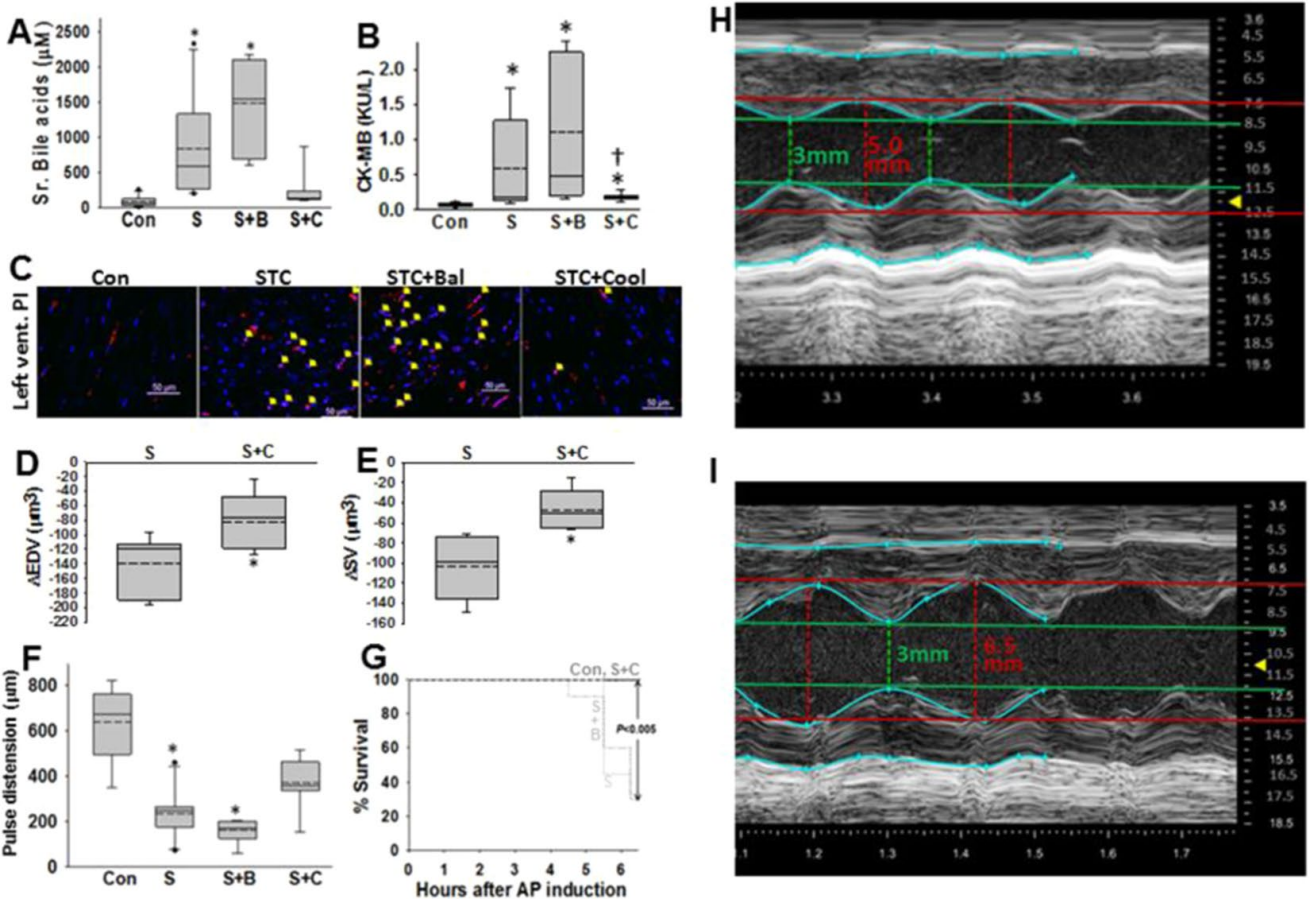
Effect of pancreatic temperatures impairing micellar breakdown on serum bile acids, cardiac injury, function, shock and mortality: (**A–F**) Samples form Control rats (Con), those with STC pancreatitis (S), STC pancreatitis with balloon and no cooling (S + B) and STC pancreatitis with cooling (S + C) were collected at the time of euthanasia. (**A)** Plasma bile acids, (**B)** CK-MB (myocardial injury marker) (**C)** Representative images showing fluorescent PI uptake (administered 5 hours into pancreatitis or when pulse distention was less than 200 μm) by heart muscle nuclei (yellow arrows). Nuclei are stained blue with DAPI. Nonspecific red fluorescence outside nuclei is from RBCs. Changes from baseline in end-diastolic volume (ΔEDV; **D**) and stroke volume (ΔSV, **E**) after 3 hours of pancreatitis in the STC vs. STC+ cooling groups. (**F)** Carotid pulse distention measured 5 hours after inducing pancreatitis. (**G)** survival curves in different pancreatitis groups. Echocardiography mages of the left ventricles of rats with STC pancreatitis (**H**), and STC+ cooling (**I**) after 3 hours, showing short axis views of the left ventricle in diastole (upper panel) and M-mode images lower panel. These were taken at the same frame size. Note the lower diastolic expansion in (**H**).

Temperatures which reduce micellar breakdown (mean 24 ± 1.5 °C) were achievable in the pancreas within an hour of initiating cooling after STC injection into the pancreas (Fig. 5A,B). These temperatures were obtained via a dual channel perfusion balloon placed in the stomach as described recently^24^. The pancreas which lies posterior to the stomach in rodents and humans was cooled in this manner while avoiding generalized hypothermia by external heating (Fig. 5A,B). The 5% STC injection^21,22^ (120 mM in saline, pH 7.4, simulating the composition of bile^31^, Fig. 1 table), followed by ligation of the biliopancreatic duct^23^ caused rapid pancreatic necrosis (18 ± 6%) in 1 hour. This necrosis was unaffected by pancreatic cooling (Supplementary Fig. 4A,B), similar to the findings with 10 mM STC *in vitro*. Similarly the acute liver injury due to ligation- which was noted as an increase in the leakage of alanine transaminase (ALT) into the serum (Supplementary Fig. 4C) we unaffected. However, pancreatic cooling reduced systemic injury and inflammation evidenced by (1) a reduction in circulating dsDNA and Histone complexed DNA fragments (Fig. 5C,D), (2) dampened increase in systemic inflammation (serum IL6 and TNF-α levels), and (3) lung inflammation (Fig. 5D–H), and also (4) improved oxygenation (Supplementary Fig. 5). To understand the mechanism underlying this protection we measured how much of bile acid had leaked from the pancreas injection into the serum. Serum bile acid levels (Fig. 6A) increased with STC infusion (831 ± 214 μM, shown as S) and were unaffected by the balloon placement (1484 ± 249μM, p = 0.34 shown as S + B vs. STC alone), but were reduced significantly (239 ± 90 μM, p = 0.007 shown as S + C vs. STC alone) by cooling the pancreas. This large reduction in serum bile acids is mechanistically consistent with what we noted in Fig. 1, and can be explained by thermodynamic interference in micellar breakdown of the infused 120 mM STC at 25 °C, thus preventing its entry into serum and distant organs where it can induce toxicity. Cardiovascular parameters were alsosignificantly improved in this manner. There was a normalization of markers of myocardial damage (CK-MB; Fig. 6B) which was associated with a reduction in propidium iodide (PI, which marks necrotic cells) positive cardiomyocytes (Fig. 6C). This translated to pancreatic cooling improving cardiac function by reducing the STC induced decrement in end-diastolic volumes (ΔEDV) and stroke volumes (ΔSV; Fig. 6D,E,H,I) while the changes in the heart rate (−20 ± 47 beats/min vs. −17 ± 30 beats/min, p = 0.8) or ejection fraction (−2% ± 18% vs. 12% ± 5% p = 0.12)were unaffected by cooling. The improvement in EDV is consistent with improved cardiac muscle sarcomere lengthening, which is energy dependent^45^, and is impaired in necrotic or dying muscle. There was also improvement in the large drop in carotid pulse distension (Fig. 6F), which signifies improved blood pressure and shock. Most significantly survival at 6 hours after induction of pancreatitis improved from 30% to 100% (p < 0.005; Fig. 6G) with pancreatic cooling.

Based on this we conclude that the improvement in outcomes is due to therapeutic pancreatic cooling to 25 °C preventing breakdown of the infused STC micelles (or STC-phospholipid mixed micelles in the refluxed bile), thus reducing circulating levels of STC, and thereby improving systemic injury, including cardiac and lung injury. This protection occurs by impairing micellar breakdown of the STC micelles injected, and is independent of the pancreatic/acinar necrosis which occurs from supramicellar STC at concentrations. Thus hypothermic interference in bile salt micellar breakdown can explain the lower serum BA levels, and reduced systemic BA toxicity, which is seen as protection from inflammation, cardiac, lung injury, and shock along with improved survival.

## Discussion

**Figure 7:**
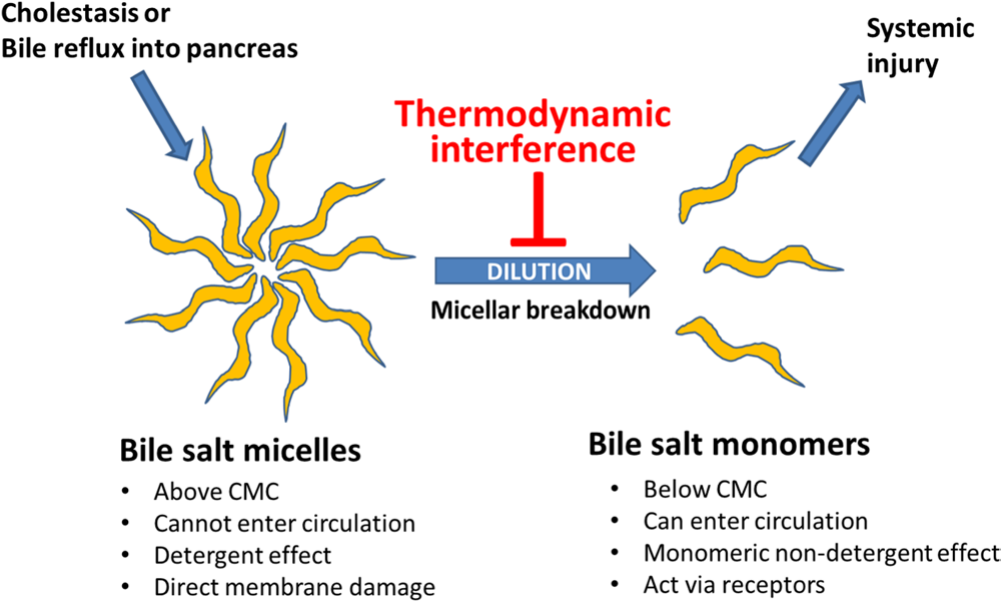
Schematic showing the effect of thermodynamic interference on micellar breakdown and consequent systemic entry and toxicity of bile salts. When there is blockage to the flow of bile, or it enters organs such as the pancreas during biliary pancreatitis, the dilution of bile salts in these abnormal environments allows for their micellar breakdown and the entry of bile salt monomers into the circulation. Targeted temperatures preventing micellar breakdown reduce the entry of these macromolecular aggregates into the circulation, reducing serum bile salts and preventing the systemic injury they induce.

In this study we learn that hypothermic interference of bile salt micellar breakdown reduces cell injury induced by monomeric bile salts, prevents their systemic entry *in vivo*, and thereby reduces systemic bile acid toxicity, which is noted as reduced inflammation and improvement in myocardial injury, shock and mortality (Figs. 5–7).

This shows that during acute cholestasis such as in the STC model of pancreatitis, Bile salt demicellization is an essential step for their entry into the circulation. Clinically, BA increase causing myocardial impairment has been noted as a lower increase in cardiac output when the heart is subjected to dobutamine stress in patients with obstructive jaundice^46^, and relief of biliary obstruction improving cardiac output and reducing markers of heart failure^47^. In our acute model, we note a drop in EDV and SV parallels the increase in serum bile acids during shock. Cooling prevents the increase in serum bile acids and the decrement in EDV and SV. These parameters are also perturbed in patients with cholestasis at risk of hepatic decompensation^48,49^, and thus may help predict organ failure. While the exact mechanisms of systemic bile acid toxicity are not known, we find evidence of cardiomyocyte injury *in vivo* along with impaired contractility (Fig. 6B–I). Previous studies have shown cardiomyocytes to have reduced contraction in the presence of bile acids^50^ and cholic acid infusion or genetic knock outs of FXR have elevated bile acids and impaired cardiac contractility^51^. Thus parameters of cardiac injury and dysfunction may help predict shock during cholestasis.

The CMC of BA has been extensively studied (summarized in^37^) and the micellar structure of STC aggregates is fairly well described in the literature. The curved, planar features of the tetracyclic steroid ring system make the assembly of STC and other bile acids fairly unique compared to traditional surfactants^52^. The hydrophobic steroid nucleus promotes^25^ self-association (aggregation) and dimerization^26^ forming primary micelles, while hydrogen bonding between hydroxyl groups of different molecules results in secondary micelles^28^. Specifically, the long hydrocarbon hydrophobic tails combined with short polar head groups for a typical surfactant, e.g., sodium dodecylsulfate, gives rise to well defined mechanisms for micelle formation. Conversely, bile acids, and STC in particular, only have 2–8 monomers in the micelle aggregate at equilibrium^53^, and below CMC can form liquid crystalline domains depending on the environment and temperature. A computational study that used molecular dynamics simulation to observe the evolution of micelle structure showed an immediate distribution of micelle sizes for taurocholate, rather than dimerization and step-wise growth^54^.

It is well known that an electric double layer and a Stern layer, comprised of the cationic counterion reside at the periphery of the micelle surface. While not 1:1, it would be inappropriate to state that counterions such as Na^+^ are not present (*in vivo* or in the buffers we use for the *in vitro* studies) and did not affect the micelle properties. Thus our findings may be relevant to both bile acids and bile salts.

Using ITC, we note that the hydrophobic effect of the steroid nucleus dominates when only a single hydroxyl is present, resulting in lower CMCs with cooling (Fig. 2C). Compared to the tri-hydroxy STC (Fig. 1A), the di-hydroxy TCDC was influenced lesser by cooling from 37 °C to 25 °C (Fig. 2B) supporting that hydrogen bonding between the molecules may stabilize micellar structure^26,28,55^. Perhaps- BA-BA or BA-solvent interaction can contribute to micellar breakdown, with cooling by making the reaction exothermic (Figs. 1A,B, 2B). However, since the magnitude of BA-solvent interaction is small (Supplementary Fig. 2A,D middle tracing), and equal to the injection of solvent into solvent, and also unaffected by temperature (Supplementary Fig. 2D); the contribution of this to micellar breakdown is below our current resolution. With three hydroxyl groups (STC), we observed a U-shaped reduction, with a minimum at ~25 °C (Fig. 1A–C), followed by an increase in CMC. We thus note 25 °C to be the cross point where detectable changes in enthalpy of STC cease over the range at which micellar transition is noted at other temperatures (Fig. 2B, Supplementary Figs. 2C, 3A,B). At this temperature, the entropic penalty for micellar breakdown is the largest (Fig. 1C), suggesting the greatest intensity of unfavorable STC-solvent interactions, which for unclear reasons was undetectable by us. As temperature is slowly decreased from 37–28 °C, we note that the CMC is not largely affected indicating that the relative concentration of micelles and monomers is unaffected by temperature in this range (Supplementary Fig. 3C). However, the decrease in titration enthalpy indicates a change in the intermolecular forces that stabilize the micelles (namely hydrogen bonding and the hydrophobic effect). As temperature decreased below 25 °C, the titration enthalpies become exothermic and the entropy changes return to the values observed at 37 °C. Based on the current data, we can conclude that the reduction in temperature increases the propensity for intermolecular hydrogen bonding, which causes the change in titration enthalpies to go from endothermic to exothermic. The role of an a small magnitude unfavorable STC-solvent interaction in the entropy change at ~25 °C, where the addition of micelles is athermal, and CMC approaches zero remains unclear. With this acknowledgement and noting our limitations, the transition we note at ≈25 °C, so far remains unexplained by the Gibbs Free Energy equation. Temperature dependent change in CMC of bile salts has not been previously reported in literature^56^. Furthermore, we use a combination of DLS and cryo-EM to quantify the size of the aggregates as well as calorimetry to understand the temperature-dependent thermodynamics of micelle formation and disassembly. Together with application-specific data to corroborate the conclusions of the mechanistic studies, we dramatically progress the understanding of bile acid assembly and its relevance to disease.

The practical result is that cooling reduces micellar breakdown of the main BAs present in rodents and humans by 60–90% (Figs. 1, 2, Supplementary Fig. 1). This reduces cellular and systemic injury (Figs. 3–6) via reducing systemic entry. The previously documented adverse outcomes from cholestasis culminating in myocardial injury^57,58^ and renal failure^10^, higher adverse events or mortality^5^ after abdominal surgery during biliary obstruction^4^, and the dismal prognosis^59^ from recurrent biliary obstruction in pancreatic cancer bring some other scenarios where impeding BA micellar breakdown may help. Application of cooling could perhaps improve outcomes in critically ill patients^9^ and those with advanced liver disease^6^ with elevated bile acids. Interestingly, while pancreatic injury from supramicellar bile, and liver injury from ligation of the biliopancreatic duct are unaffected by cooling (Figs. 3C,D, 4D,E, supplementary Fig. 4), similar findings with the mixed micellar system of STC+ POPC (Fig. 4), along with the protection we note in cell and animal models support the need to study this phenomenon in more detail in more complex mixed micellar systems. The case reports of ABCB4 mutations^60^ resulting in cholestasis and recurrent (but not severe) biliary pancreatitis, support that the lack of phophatidyl choline may increase the risk of developing pancreatitis, but not its severity. Therefore further studies using more complex micellar systems are unlikely to affect our conclusions.

Our studies are limited by being acute and not accounting for the change in microbiome that may take place after prolonged cholestasis lasting several days or months. However these do support the role of systemic entry of bile acids in worsening systemic injury and survival.

In summary, we show that breakdown of Bile salt micelles is essential for their entry into the circulation and that cooling can significantly reduce this. This reduction in circulating bile acids can ameliorate cholestasis induced inflammation, lung injury, myocardial dysfunction, shock and death.

## Materials and Methods

All experimental protocols were approved by the Institutional review board (IRB) and the Institutional animal care and use committee (IACUC) at Mayo Clinic. The studies were carried out in accordance with relevant guidelines and regulations. These are described below.

### Reagents

Bile acids including sodium salts of taurolithocholic acid (TLCA), taurochenodeoxycholic acid (TCDC) and Taurocholic acid sodium (STC; Sigma, St. Louis, MO, USA) were prepared in sterile saline or phosphate buffered saline (pH 7.4) as was 1-palmitoyl-2-oleoyl sn-glycero-3-phosphocholine (POPC, 9:1 ratio, Avanti Polar Lipids Inc, Alabaster, AL) *In vitro* these were freshly made with sonication unless described otherwise. Propidium Iodide was from (PI; Sigma, St. Louis, MO, USA). Isoflurane USP was from Piramal Critical Care INC, Bethlehem, PA, and was used for sedation as described previously^24^.

### *In vitro* studies

Acini were harvested from mice with IACUC approval. All data shown are from ≥3 independent experiments.

### Pancreatic acini harvest

Acini were made from 8 to 10 weeks old male CD-1 (Charles River Laboratories, Wilmington, MA) as described previously^13,24,61–63^. Viability was >95% cell viability was by Trypan blue exclusion prior to use.

### Lactate dehydrogenase assay

As previously^13,24,62,63^, acini in HEPES buffer with 0.01% BSA were exposed to STC (Sigma, St. Louis, MO) at the indicated concentrations and temperatures (15 °C to 37 °C) in a shaking (60RPM) water bath. Supernatant was removed at the indicated time point, and the LDH assay was carried out with Cytotoxicity detection kit (Roche, Mannhein, Germany) as per manufacturer instruction. A total of 4–8 independent experiments with duplicate were performed.

### Intracellular Calcium imaging and Mitochondrial depolarization measurement

This was done as described previously^24,62,63^ in acini loaded with fura-2AM (5 µg/ml; Molecular Probes, Invitrogen) and 5,5′,6,6′-Tetrachloro-1,1′,3,3′-tetraethylbenzimidazolylcarbocyanine iodide (JC-1, 5 µg/ml; Enzo Life Sciences, Farmingdale, NY) at 37°C for 30 min in HEPES buffer with 0.1% BSA. After loading, the cells were washed and kept on ice until use at 37°C. Studies were done in suspensions of cells in a quartz cuvette of a F2100 Hitachi Fluorescence Spectrophotometer. Cell were stimulated with different chemicals (e.g. STC, EGTA, Thapsigargin) at the indicated concentrations. Changes in intracellular calcium concentrations were determined by alternately exciting at 340 nm and 380 nm, and measuring emission at 515 nm. Mitochondrial inner membrane potential (Ψm) were determined by excitation at 488 nm, and alternate measuring emission at 530 and 590 nm using the A decrease in the JC-1 aggregate fluorescence at 590 nm and an increase in the fluorescence at 530 nm reflect depolarization. As described previously^24,62,63^, the emission ratio measured at 515 nm after exciting at 340/380 (for Fura-2AM), and 530/590 emission ratio (after exciting at 488 nm, for JC-1) were used. Data presented are means ± SD from at least three independent experiments.

### Isothermal titration calorimetry (ITC)

As described previously^24,62^, the experiments were carried out on a Nano ITC (TA Instruments-Waters LLC, New Castle, DE) instrument using Nano ITCRun Software v3.3.0, equipped with an automated micro-syringe injection device which allowed injecting small amounts of stock solution (increments of 0.1 µl) into the sample chamber. For demicellization experiments, a total of 55 aliquots of degassed STC (50 mM, 0.85 µl per injection) were injected from a rotating syringe (350 rpm) into the ITC sample chamber containing buffer equilibrated at 25 °C, 28 °C, 31 °C, 34 °C and 37 °C. Double distilled water was used in reference electrode chamber. The interval between two injections was kept at 200 s. The control experiment was performed injecting PBS into PBS or bile acid into PBS in a similar set up. To check the effect of POPC on STC demicellization a total of 40 aliquots of degassed STC or STC with POPC (9:1 ratio, 250 mM, 0.85 µl per injection) were injected similarly from a rotating syringe (250 rpm) into the ITC sample chamber at 25 °C and 37 °C. The interval between two injections was kept at 300 s. The ITC raw data were analyzed by using NanoAnalyze v2.4.1 software.

ΔH was calculated by averaging the initial and final titration enthalpies. A corrected value to account for the titration of monomer was calculated by multiplying by C_T_/(C_T_ − CMC), where C_T_ is the STC concentration In the syringe (50 mM) and CMC is the measured CMC from the inflection point of the titration curve. The inflection point was determined using the derivative method, where sigmoidal titration curves were fitted with a 3^rd^ order polynomial, which was differentiated. As described previously^64^, the change in the Gibbs Free Energy was calculated using the following expression:

$$\Delta {G}_{mic}^{0}=(2-\alpha )RTln(cmc)$$

Where *α* is the degree of ionization, R is the universal gas constant, and T is the absolute temperature^64^. The degree of ionization was taken to be 0.87 based on previous literature^37,65^. Finally, values for -TΔS were calculated using ΔG = ΔH − TΔS.

### Dynamic light scattering (DLS)

This was done based on previous protocols^66,67^ on a Malvern Zetasizer NanoZS (Malvern Panalytical Ltd, UK) having a a 4 mW 633 nm He/Ne laser. Samples equilibration time for temperature was 15 min before analysis. The CONTIN algorithm^66^ was used to fit raw autocorrelation data along with the L-curve criterion to select an optimal regularization parameter^67^. MATLAB scripts for the above algorithms, written by Marino and Hansen were combined and utilized for data analysis. Data were collected in triplicate and averaged with error bars representing point standard deviation.

### Cryogenic electron microscopy

Carbon-coated copper (Quantifoil R 2/1) grids were plasma cleaned for 60 s and transferred to a FEI Vitrobot held at 25 °C and 100% relative humidity. A 4 µL aliquot of solution was pipetted onto the grid inside the chamber. Excess liquid was removed by blotting once with filter paper for 6 s. After plunging into liquid ethane, the samples intended to be imaged were stored in liquid nitrogen. This was consistent across all samples. Samples were imaged on a Tecnai F20. This was operated at a high tension of 200 kV. Images were collected using a Gatan Orius SC200–830.20B camera with a nominal underfocus of around 3 µm to improve contrast. The microscope and grid holder were kept sufficiently cooled to liquid nitrogen temperatures while imaging.

### Human samples

As described previously^13,68^, waste, deidentified fluid from pancreatic endoscopic of surgical procedures for biliary pancreatitis associated necrosis was collected as a part of an approved IRB protocol^13^. As per the protocol approved by the IRB, informed consent was obtained from all relevant subjects. 14 samples were analyzed except for 1 sample which was from a disrupted pancreatic duct. As recommended, the collections were more than 4 weeks old at the time of draininge^69,70^. Samples, transported at room temperature or shipped overnight, were spun for 5 minutes at at 300 g, the supernatant was sonicated and frozen at −80 °C for subsequent analyses after a single thaw on ice. This fulfills the Biospecimen Reporting for Improved Study Quality (BRISQ) criteria reporting guidelines^71^.

### Cooling balloon design

This is as described previously^24^.

### Animal work, surgery and cooling experiments

This was as described previously^24^ as per IACUC approval. All animal experiments were approved and carried out in accordance with the Institutional Animal Care and Use Committee (IACUC) at Mayo Clinic (Scottsdale, AZ). Animals, [Male Wistar rats of 350–400 grams (Charles Rivers Laboratories, Wilmington, MA)], fed standard laboratory chow, housed with a 12-h light/dark cycle, were allowed to drink ad libitum and fasted overnight. There were atleast 8 animals/group. The surgery, balloon placement, temperature monitoring thermocouple placement are described previously^24^. Using the technique described previously^24^, severe AP was induced by intra pancreatic duct retrograde injection of 5% sodium taurocholate solution (50 µl/100 mg of body weight) followed by ligating of the bilio-pancreatic duct just proximal to its entry into the duodenum. Post-operative care and balloon infusion with cold water was as described previously^24^. Cooling was started after the infusion of STC into the pancreas. It took 30 minutes to 1 hour to achieve target temperatures (Fig. 5B), and thus the cooling was therapeutic. Generalized hypothermia was avoided by monitoring rectal temperatures and external heating as described previously^24^. Vital monitoring, hydration and euthanasia (6 hours after STC infusion) are as described previously^24^

### Echocardiography

Rats were sedated with isoflurane 1–3%, shaved and hair was removed from the ventral thorax using a depilatory cream. Heart function was evaluated by echocardiography using high-frequency small animal ultrasound system (Vevo 3100, FUJIFILM VisualSonics Inc., Toronto, ON, Canada). This had a 15–30 MHz center frequency linear transducer (MX250, FUJIFILM VisualSonics Inc.). The transducer was positioned parallel to the short and long axis of the Left ventricle (LV). Images were acquired and using the Vevo LAB analysis software (v3.0). M-mode views of the heart were used to outline the endocardial and epicardial borders of the LV. The LV chamber was defined by the endocardial border. The LV myocardium was the space between the and the endocardial and epicardial borders. Heart rate (bpm); LV end diastolic and systolic diameter (mm); stroke volume (µl); diastolic and systolic volume (µl); Ejection Fraction (%); LV Anterior Wall diastolic and systolic thickness (mm); LV Posterior Wall diastolic and systolic thickness (mm) were measured at M-mode in both short and long axis for at least 2 consecutive measurements, before and 3 hours after AP induction.

### Bile acid composition

These were measured by LC-MS/MS at Mayo Medical Labs as described previously^13^.

### Biochemical assays

The assays were done as previously^24^ per the manufacturer protocol (amylase, lipase: Pointe Scientific, Canton, MI; Total Bile Acids: BQKits, San Diego, CA). Tests were performed on a GloMax® Multimode Detection System (Promega).

### Cytokine/chemokine assays

plasma cytokine/chemokine protein levels were assayed as previously^24^, with a MILLIPLEX MAP Rat Cytokine/Chemokine Magnetic Bead Panel Assay (Millipore) according to manufacturer’s recommendations on a Luminex 200 System (Invitrogen) and analyzed using the xPONENT software.

### DNA damage

As previously^24^, Quant-iT™ PicoGreen® dsDNA Reagent (Invitrogen) was used for quantification of double-stranded DNA and Cell Death Detection ELISA kit (Roche) used to quantify cytoplasmic histone-associated DNA fragments (mono- and oligonucleosomes) in plasma.

### Histology and immunofluorescence staining

As previously^24^, the pancreas, Kidney and lung tissue were fixed with 10% Buffered Formalin, Phosphate Buffer and processed, embedded in paraffin. Sections (5 μm) were used for hematoxylin and eosin (H&E) staining and for Myeloperoxidase (MPO) immunofluorescent staining as described previously^24^. In brief, after deparaffinization and antigen epitope retrieval, tissue were blocked in 5% Normal Goat Serum tissues (Sigma, St. Louis, MO) in PBS for 30 min at room temperature followed by incubation overnight at 4 °C with the primary rabbit polyclonal antibody against MPO (1:50, Abcam, Cambridge, MA). Excess antibody was removed by rinsing in PBS followed by application of secondary fluorescently labeled Goat anti-Rabbit IgG (H + L) Alexa Fluor® 750 conjugate (1:100, Life technologies, Carlsbad, CA) at room temperature for 1 hour. The staining was completed with fluorescent nuclear counterstain with Hoechst 33342 solution 20 mM (1:1000, Thermo Scientific). Digital images of section were captured with a digital microscope Axio Imager.M2 or Axio Observer.Z1 (Zeiss, Oberkochen, Germany). MPO positivity was quantified as percentage of stained area to total nuclear area in the lung tissue, both calculated by color thresholding using a uniform counting algorithm on ImageJ. 5 images per organ per animal were obtained and quantified as described previously^24^. The algorithms were are as described previously^24^.

### Visualization of cell death

For labeling of necrotic cells in heart Propidium Iodide (5 mg/kg body weight, diluted in 150 µL saline; injected 1 hour before experiment end point) was I.V. injected. Tissue slides counterstained with DAPI were imaged using a digital microscope.

### Pancreatic necrosis

As described previously^24^, whole pancreas H&E-stained sections were examined by a trained morphologist blinded to the sample. Briefly, all pancreatic parenchymal area was imaged using the PathScan Enabler IV slide scanner (Meyer Instruments, Huston, TX) and images were evaluated for acinar necrosis. Necrotic area and total acinar area were measured in pixels for each pancreas. Percentage necrosis was reported as a percentage of total area for each pancreas.

### Statistical analysis and graphical representation

As described previously^24^, independent variables for *In vivo* studies are shown in Box plots in which mean (dotted line), median (solid line), *In vitro* data is shown as bar graphs reported as mean ± SD. line graphs were used for continuous variables. All values are reported as mean ± SD. Significance levels were evaluated at p < 0.05. Data for multiple groups were compared by 1-way ANOVA versus controls and values significantly different from controls were shown as (*). When comparing 2 groups a t-test or Mann-Whitney test was used depending on the normality of distribution and shown as (ǂ) when different from control. When a therapeutic intervention (e.g. cooling balloon) was different from controls, it was compared to the placebo treated (balloon only) group. Significant differences were denoted as (†). Graphing was done using SigmaPlot 12.5 (Systat Software, Inc, San Jose, CA).

## Supplementary information

Supplementary Data.
