# Supplementary material for: Thermodynamic interference with bile acid demicelleization reduces systemic entry and injury during cholestasis
Source: Sci Rep. 2020 May 21;10:8462. doi: 10.1038/s41598-020-65451-w (PMC7242474; doi:10.1038/s41598-020-65451-w)
Supplement: Supplementary file 1 — Supplementary Data. [file 41598_2020_65451_MOESM1_ESM.pdf]

Thermodynamic interference with bile acid demicelleization reduces systemic entry and injury during cholestasis

Short title: Interference with bile acid micellar breakdown reduces systemic entry.

Cristiane de Oliveira[1*], Biswajit Khatua[1*], Bara El-Kurdi[1], Krutika Patel[1], Vivek Mishra[2], Sarah Navina[3], Bradley J. Grim[4], Srishti Gupta[4], Marek Belohlavek[5], Brian Cherry[6], Jeffery Yarger[6], Matthew D. Green[4], Vijay P. Singh[1,7]

[1]Department of Medicine, Mayo Clinic, Scottsdale, AZ, Department of Medicine[2], Pathology[3], University of Pittsburgh, Pittsburgh, PA. , [4]Department of Chemical Engineering, School for Engineering of Matter, Transport and Energy, Arizona State University, Tempe AZ. [5]Department of Cardiovascular Medicine Mayo Clinic Arizona. [6]School of Molecular Sciences, Arizona State University, Tempe AZ [7]Department of Biochemistry and Molecular Biology, Mayo Clinic Arizona.

** Contributed equally to the study*

Grant support: Supported by Grant number PR110417 Award # W81XWH-12-1-0327 from the Department of Army (DOA) (VPS), award number R01DK092460, R01DK119646 (VPS) from the National Institute of Diabetes and Digestive and Kidney Diseases (NIDDK). The funders had no role in study design, data collection and analysis, decision to publish, or preparation of the manuscript. The contents of the manuscript are solely the responsibility of the authors and do not necessarily represent the official view of DOA, NIDDK. Also supported by a startup package from the Mayo Clinic Arizona, Department of Medicine (VPS). This work was supported in part by funds through the 2019 ASU-Mayo Seed Grant (MG). MG recognizes the 2018 Mayo Clinic and ASU Alliance for Health Care Summer Residency Fellowship as well as NSF CBET-1836719, the Army Research Office (W911NF-18-1-0412), and NASA (80NSSC18K1508) for funding.

Disclosures: No conflicts of interest exist.

Author contributions: VPS designed and conceptualized the study. Acquisition of data was carried out by CD, BK, BEK, KP, SN, VM, BG, SG, MG and VPS. Statistical analysis was done and manuscript was drafted and revised for important intellectual content by CD, BK, BEK, BG, BC, JY, MB, MG, and VPS. Funding was obtained by MG, and VPS and the entire study was supervised by VPS

Corresponding Author:
Vijay P. Singh, MD,
Division of Gastroenterology and Hepatology
Mayo Clinic,
Scottsdale, AZ 85259,
Phone: 480-301-4286
Fax: 480-301-7017
Email: singh.vijay@mayo.edu

SUPPLEMENTARY FIGURES

Composition of human biliary pancreatitis collections

Bile acid in human biliary AP	Percentage
Chenodeoxycholic Acid	35.3±3.5
Cholic Acid	37.7±5.8
Deoxycholic Acid	15.0±3.8
Hydroxycholestanoic Acid	1.9±0.8
Hydrodeoxycholic Acid	0.9±0.3
Lithocholic Acid	0.6±0.2
Ursodeoxycholic Acid	8.5±2.5

Cholic acid conjugates in human biliary AP	Percentage
Cholic Acid	9.3±11.1
Glycocholic Acid	13.3±14.5
Taurocholic acid	19.2±18.7

Supplementary figure 1: Tables showing the bile acid composition of human biliary pancreatitis fluid collections. The conjugates of the most prevalent one in each group are mentioned in the table to the right. Note cholic acid conjugates form the most predominant bile acids.

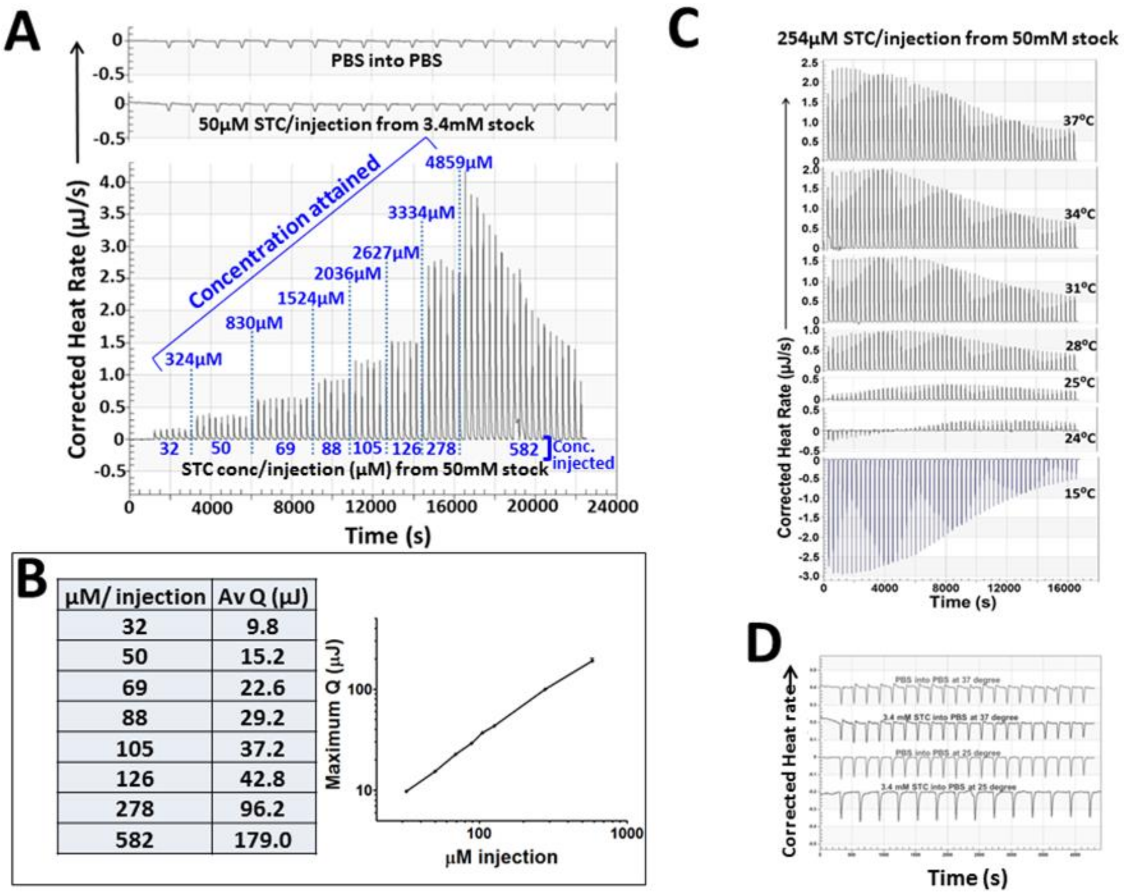

μM/ injection	Av Q (μJ)
32	9.8
50	15.2
69	22.6
88	29.2
105	37.2
126	42.8
278	96.2
582	179.0

Supplementary figure 2: A) isotherms at 37°C showing injection of PBS (pH, 7.4, containing 150mM Na) into PBS (top tracing), 50μM/injection from a 3.4 mM STC (sub-micellar) stock in PBS into PBS (middle tracing), and bottom tracing showing 50 mM STC stock in PBS (which is 8-10x published CMC) injected at progressively increasing concentrations listed below the tracing in blue (Conc. Injected). The concentrations in blue (shown obliquely) are those achieved at the end of each set. Note the stable heat rate for each concentration till 4859μM is achieved, after which the magnitude of heat rate progressively decreases with no further micellar breakdown. **B)** Table and graph showing the average heat change as a function of concentration of STC/injection. Note the linear relationship. **C:** Thermograms representative of micellar breakdown of STC at the indicated temperatures. Note the progressive reduction in magnitude of heat rate and transition from endothermic to exothermic at 15C.**D:** isotherms at

37°C (top 2) and 25°Cshowing injection of PBS (pH, 7.4, containing 150mM Na) into PBS (top tracing), and 50μM from a 3.4 mM STC (sub-micellar) stock in PBS.

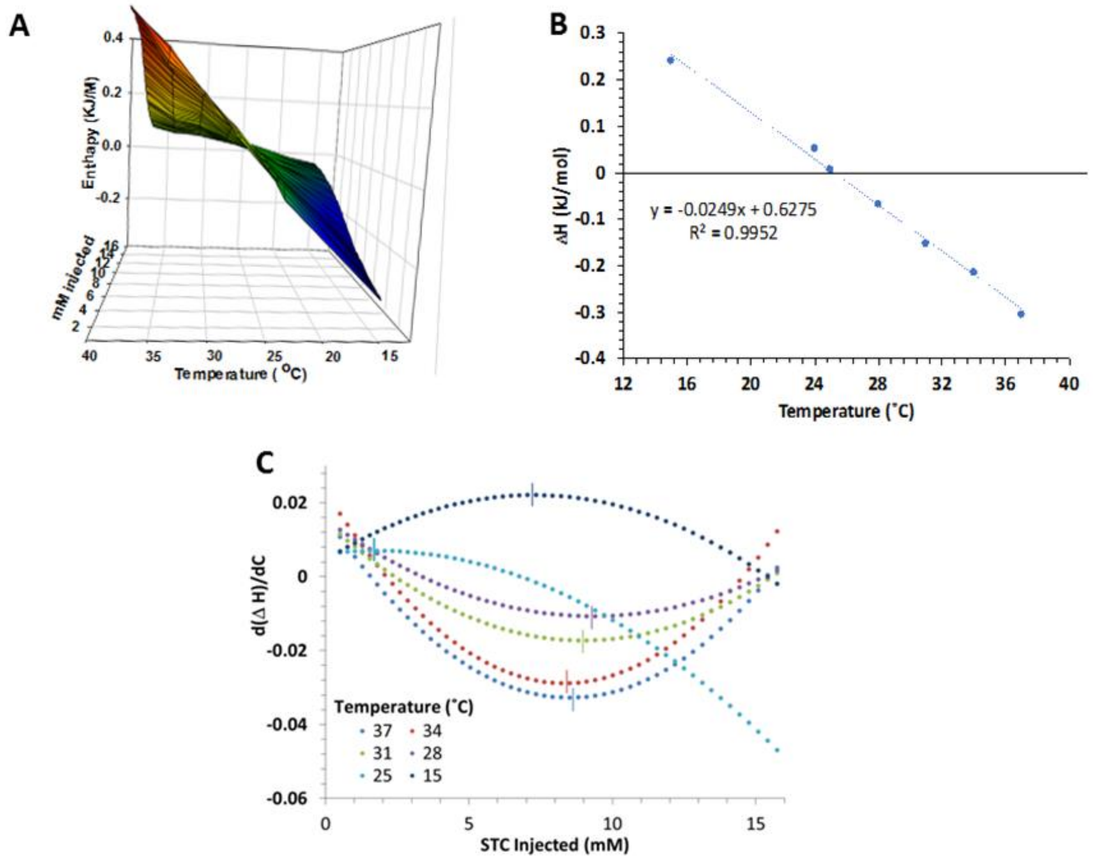

Supplementary figure 3: A: Graph showing change in enthalpy of STC micellar breakdown over the range of concentrations till equilibrium was reached, shown here as a function of temperature. Note that the lines intersect at 25C when there is no change in enthalpy on diluting STC micelles. **B:** Change in enthalpy (ΔH) from start to finish of the ITC titration at various temperatures. The linear relationship with temperature suggests an athermal titration occurs at ~25 ˚C, which corresponds with a reduction in CMC and stable micellar aggregates of STC. **C)** The derivative of enthalpy with respect to STC concentration titrated is plotted versus STC concentration injected. The maximum or minimum of the curves indicates the CMC, which is shown as a small vertical bar. The CMC remains largely stable at all temperatures except 25 ˚C.

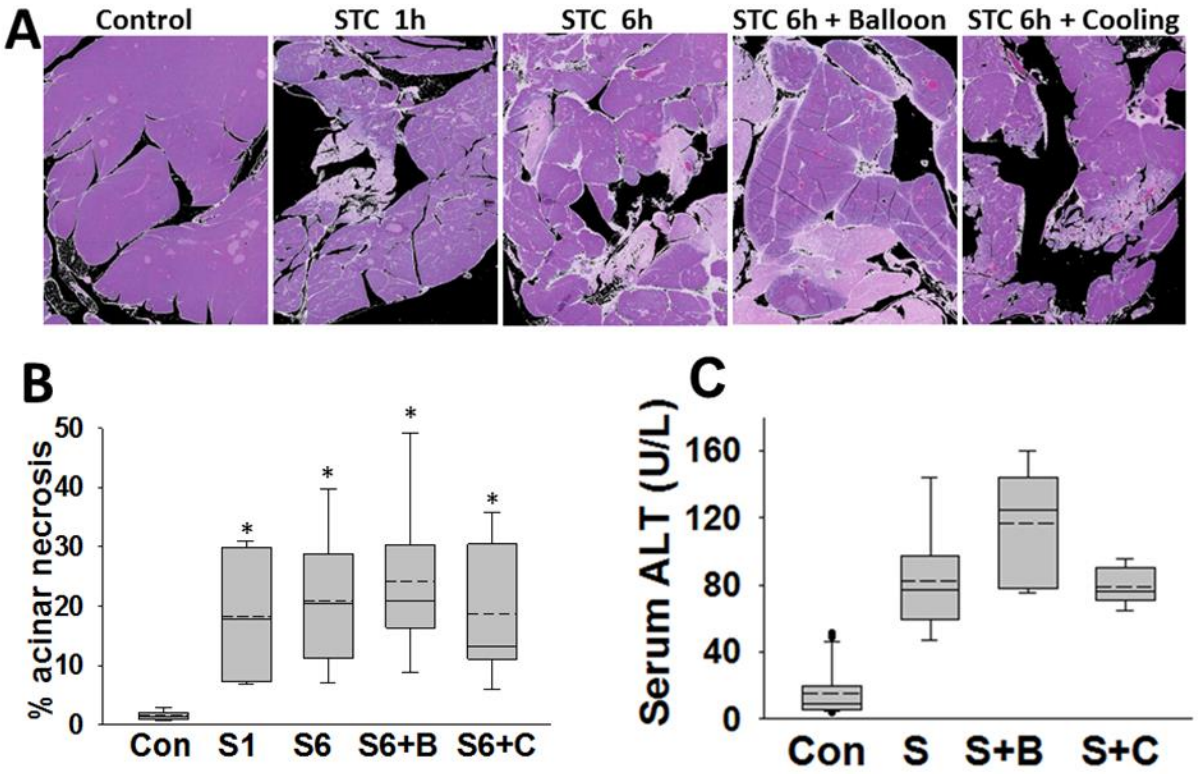

Supplementary figure 4: A, B: Pancreatic necrosis, seen as pale pink areas in A during STC pancreatitis. Histologic appearance (A) and its quantification (B) during STC pancreatitis. S1 and S6 denote necrosis after 1 and 6 hours of STC infusion alone, or 6 hours with a balloon alone (S6+B) or with balloon cooling to 25°C (S6+C). **C:** Serum ALT in the different groups at the time of euthanasia.

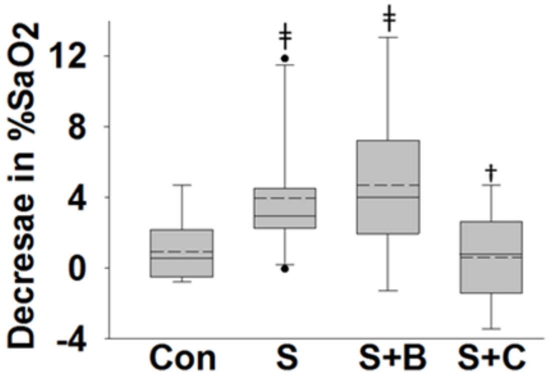

Supplementary figure 5: Box plots showing the reduction in oxygen saturations (%SaO2) from the baseline collected at the time of anesthesia and within an hour of inducing pancreatitis. B denotes balloon and C cooling to 25°C. ‡ denotes a significant difference from controls (Con) and † denotes a significant difference between the balloon and cooling groups. The %SaO2 in the cooling group remained significantly higher when compared to the pancreatitis groups without cooling.
